# Treating Bladder Cancer: Engineering of Current and Next Generation Antibody-, Fusion Protein-, mRNA-, Cell- and Viral-Based Therapeutics

**DOI:** 10.3389/fonc.2021.672262

**Published:** 2021-05-27

**Authors:** Jan P. Bogen, Julius Grzeschik, Joern Jakobsen, Alexandra Bähre, Björn Hock, Harald Kolmar

**Affiliations:** ^1^ Institute for Organic Chemistry and Biochemistry, Technical University of Darmstadt, Darmstadt, Germany; ^2^ Ferring Darmstadt Laboratory, Biologics Technology and Development, Darmstadt, Germany; ^3^ Ferring Pharmaceuticals, International PharmaScience Center, Copenhagen, Denmark; ^4^ Global Pharmaceutical Research and Development, Ferring International Center S.A., Saint-Prex, Switzerland

**Keywords:** antibody engineering, protein engineering, bladder cancer, urothelial carcinoma, ADC, immunotherapy, immuno-oncology, recombinant viral vector vaccines

## Abstract

Bladder cancer is a frequent malignancy and has a clinical need for new therapeutic approaches. Antibody and protein technologies came a long way in recent years and new engineering approaches were applied to generate innovative therapeutic entities with novel mechanisms of action. Furthermore, mRNA-based pharmaceuticals recently reached the market and CAR-T cells and viral-based gene therapy remain a major focus of biomedical research. This review focuses on the engineering of biologics, particularly therapeutic antibodies and their application in preclinical development and clinical trials, as well as approved monoclonal antibodies for the treatment of bladder cancer. Besides, newly emerging entities in the realm of bladder cancer like mRNA, gene therapy or cell-based therapeutics are discussed and evaluated. As many discussed molecules exhibit unique mechanisms of action based on innovative protein engineering, they reflect the next generation of cancer drugs. This review will shed light on the engineering strategies applied to develop these next generation treatments and provides deeper insights into their preclinical profiles, clinical stages, and ongoing trials. Furthermore, the distribution and expression of the targeted antigens and the intended mechanisms of action are elucidated.

## Introduction

The genitourinary system encompasses reproductive organs and the urinary system. The latter comprises the kidneys, which are being connected to the bladder in the lower pelvis *via* the ureters. The bladder is a hollow organ, which releases upon muscle contraction urine *via* the urethra. Bladder cancer (BC) is the second most common genitourinary malignant disease and the 10^th^ most common cancer, causing nearly 570.000 new cases and 210.000 deaths worldwide each year (1, 2). It typically affects older adults, and peak incidence occurs in the seventh and eighth decades of life. Males are four times more likely to develop bladder cancers. Hence BC is the sixth most common cancer in males and the ninth leading cause of cancer-related deaths (2).

Bladder cancer is categorized by cell type and by stage of tissue invasion. Besides urothelial carcinoma/transitional cell carcinoma (UC/TCC), which is by far the most common type of bladder cancer (90-95%), other malignancies as squamous cell carcinoma (2-5%), adenocarcinoma (0.5–2%), and small-cell carcinoma (<1%) all describe tumors of the bladder (3). Even though UC/TCC predominantly occurs in the bladder, the ureters and the urethra can be affected as well.

**Figure 1:**
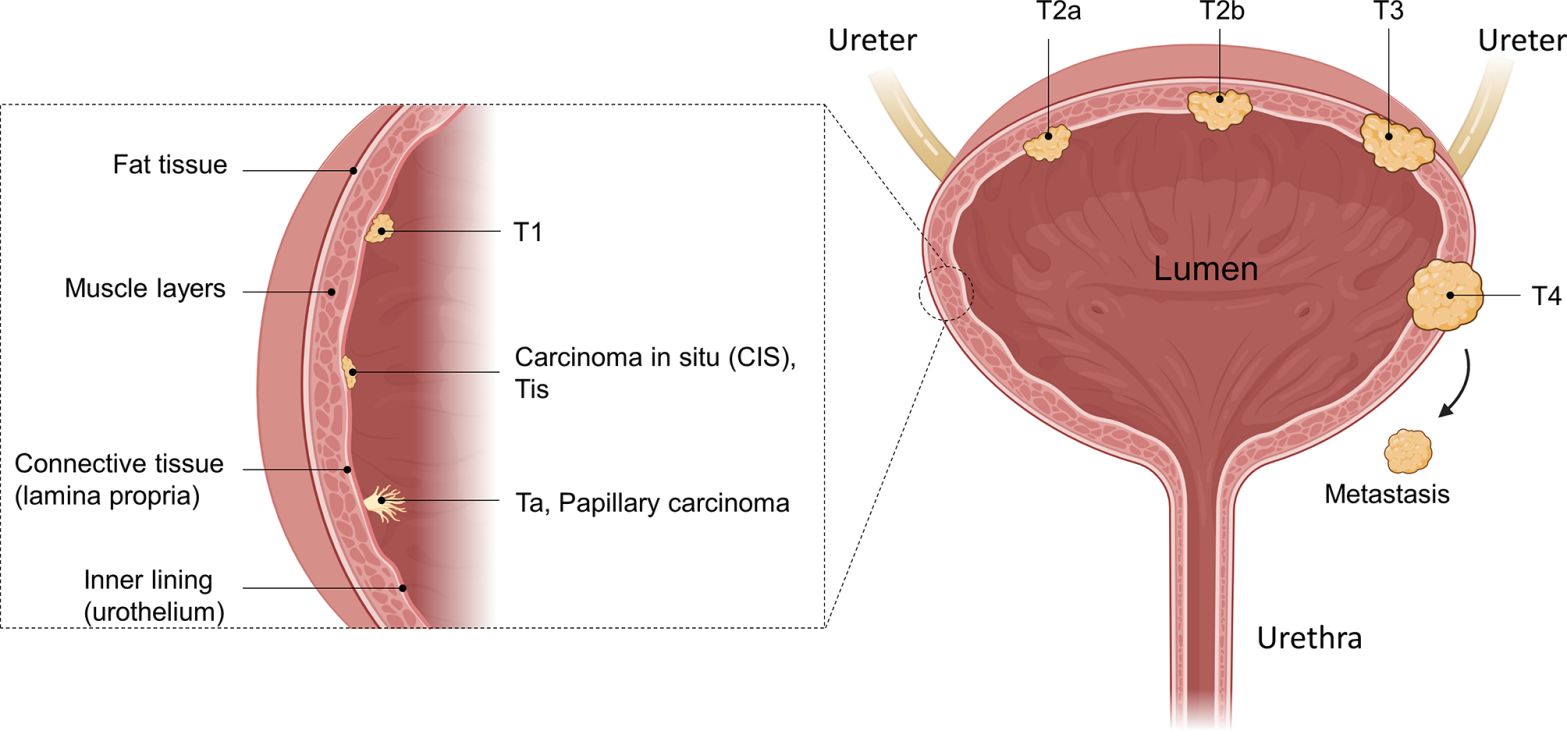
Anatomical illustration of different bladder cancer stages. The bladder as a whole, as well as a zoomed-in illustration of the bladder wall is shown. Different tumors in different tumor stages are illustrated as well as their invasion into the muscle tissue. While Ta, Tis, and T1 are defined as non-muscle-invasive, muscle-invasive bladder cancer (MIBC) disease begins with T2. After penetrating the muscle and reaching the fatty tissue, the T3 stage is reached, where T4 is characterized as growth of the tumor into other organs or metastasizing. Created with BioRender.com.

In general, UCs are classified as either non-muscle-invasive bladder cancer (NMIBC), which accounts for approximately 75% of cases, or muscle-invasive bladder cancer (MIBC), which is found in approximately 25% of patients (4, 5). Both are further staged using the Tumor, Node, Metastasis (TNM) classification. This describes how far a tumor has grown (T), whether the cancer has spread to nearby lymph nodes (N) and whether it has started to metastasize (M). Ta describes a non-invasive papillary carcinoma confined to the mucosa growing from the inner lining (urothelium) into the lumen of the bladder (**Figure 1**). Tumors growing flat on the urothelium, not invading the connective tissue, and not extending into the lumen are classified as carcinoma *in situ* (CIS), known as Cis/Tis. Papillary tumors invading the lamina propria and the connective tissue are classified as T1. Ta, Tis, and T1 are classified as NMIBC, while stages ≥ T2 are classified as MIBC. In T2a or T2b, the cancer has invaded the inner or the outer detrusor muscle, and at T3, the tumor has invaded beyond the muscle layer and the surrounding fat tissue. At T4, the tumor has begun to grow through the bladder wall into the pelvic or abdominal wall, invading adjacent organs and/or started to spread to nearby lymph nodes or even to more distant organs (metastasis, M1) (6).

This staging system was created by the American Joint Committee on Cancer (AJCC) and the International Union Against Cancer (UICC). Histological grading follows the 2004 World Health Organization (WHO) grading system for flat and papillary lesions (4). The system was an upgrade to the previous system from 1973, which uses grades 1, 2, and 3 and may still be used. In 2016 the WHO further improved the classification system to the most recent version (7). Today tumors can be classified as low grade or high grade. While the previously defined grade 2 can be classified as low or high grade, CIS belongs to high grade.

Today, the diagnosis of bladder cancer is performed more and more utilizing molecular analysis (8, 9). Genetic profiling allows for the subdivision of tumors into the basal and luminal subtype, which has a distinct impact on the success of chemotherapeutic treatment (10–12). Following diagnosis and tumor staging, NMIBC tumors are stratified by risk of recurrence and/or progression: While low-grade tumors consist of slowly growing, more differentiated cells, high-grade tumors are poorly differentiated and tend to spread faster. Furthermore, low-grade tumors have a lower risk of recurrence, while high-grade tumors have a higher risk of progression and metastasizing (6). Physicians have multiple options for diagnosing of UCs. However, cystoscopy is considered the key diagnostic tool for bladder cancer. To evaluate the cell type and stage of tissue invasion, a Transurethral Resection of the Bladder (TURB) is performed to confirm the diagnosis and stage the patient correctly.

All papillary tumors are resected completely during the TURB, which in some cases can be considered a cure. CIS cannot be resected completely during the TURB and thus need additional instillation therapy to reduce tumor burden, recurrence, and/or progression (4). For high and intermediate risk NMIBC patients, there is evidence of significantly reducing recurrence rate by additional instillation therapy to the bladder of either a chemotherapeutic agent, like mitomycin c, or Bacillus Calmette Guérin (BCG). Single instillation (SI) of mitomycin c (postoperative), a potent chemotherapeutic agent which crosslinks DNA, has been shown to reduce the 5-year recurrence rate by 14% (59%-45%) (13). For most NMIBC patients, intravesical immunotherapy utilizing BCG is the standard of care (14, 15). BCG is a non-pathogenic bacillus derived from *Mycobacterium bovis* and represents the only available vaccine for tuberculosis (16). Most of the time, BCG treatment starts after TURB, aiming at preventing tumor recurrence (17).

Although failure after BCG instillation therapy is observed in 30% to 40% of cases (18, 19), patients who experience early failure are defined as having BCG unresponsive NMIBC disease (20, 21). These patients are very unlikely to benefit from further BCG therapy and are recommended radical cystectomy, which describes the complete removal of the bladder. The primary treatment for patients with MIBC depends on the M (metastasis) status. For localized MIBC (M0), the primary and standard treatment is the complete removal of the bladder and creation of a urinary diversion (radical cystectomy) +/- neoadjuvant therapy. In very select cases, a bladder-sparing approach can be adopted. Controversies exist regarding age and type of diversion (5). Non-eligible patients can be offered radiotherapy. 10-15% of patients with muscle-invasive disease are already metastatic at diagnosis (22). As first-line treatment in advanced stages, platinum-based chemotherapeutics, like cisplatin, are utilized, which interfere with DNA replication in fast-growing cells (23). Furthermore, intravenous application of the nucleoside-analog gemcitabine, structurally related to cytidine, and instillation of mitomycin c are chemotherapeutic treatment options (24). Even though bladder cancer is relatively chemosensitive, reoccurrence after first-line treatment with the chemotherapeutics gemcitabine plus cisplatin results in poor prognosis (25). Resistance mechanisms include the elevated drug efflux as well as reduced influx. Furthermore, increased repair of the DNA leads to drug resistance. Multiple enzymes and repair mechanisms, including ERCC1, PARP, Nrf2, CTR1, are involved in the development of drug resistance (26). A detailed review on resistance of bladder cancer against conventional therapies can be found elsewhere (26, 27). If treatment fails, it might become necessary to perform cystectomy, where the bladder is partly or completely removed (28). As this is a radical measure with a significant impact on the quality of life for respective patients, new treatment options are necessary, allowing for a more precise treatment and circumventing side effects that are observed in classical chemotherapies.

Immunotherapy approaches revolutionized cancer therapy in the last decades. It is based on the concept of activating the patients’ immune system to eradicate the malignant cells. Even though a successful biotherapy applied for approximately 40 years, the anti-tumor mechanisms associated with BCG are not fully understood (29). Nevertheless, BCG-mediated effects are presumably based on the involvement of CD4+ and CD8+ lymphocytes, natural killer (NK) cells, and granulocytes, as well as a variety of cytokines (29). As BCG-failure is observed in 30% to 40% of NMIBC patients (18, 19), there is a need for new immunotherapy-based drugs. Different engineering strategies and antibody formats were investigated in (pre-)clinical settings over the last decades to employ new mechanism of actions. On the one hand, novel approaches based on antibody engineering like monoclonal antibodies (mAbs), antibody-drug-conjugates (ADCs), and bispecific molecules have been explored. On the other hand, gene therapeutics, mRNA- and cell-based therapies are emerging as promising tools to treat urologic cancers. Here we review those engineering strategies, illuminate the mechanism of action, and discuss clinical trials (**Table 1**).

**Table 1:** Overview of clinical trials targeting bladder cancer with engineered biologics.

	Drug (Target)	NCT Number	Combined with (or compared to)	Disease	Number of patients (estimated or actual enrolled)	Phase	Status
**Monoclonal Antibody**	Lirilumab (KIR2DL)	NCT03532451	Nivolumab	Bladder Cancer	43	I	Active, not recruiting
	Vofatamab (FGFR3)	NCT02401542	Docetaxel	Locally Advanced or Metastatic Urothelial Cell Carcinoma, Urinary Bladder Disease, Urological Diseases	71	I/II	Terminated (program has been put on hold by the sponsor)
		NCT02925533	Pembrolizumab	Bladder Cancer	1	I	Terminated (Terminated due to safety concerns.)
		NCT03123055	Pembrolizumab	Locally Advanced or Metastatic Urothelial Cell Carcinoma, Urinary Bladder Disease, Urological Diseases	28	I/II	Terminated (program has been put on hold by the sponsor)
**Bispecifc Antibody**	Orlotamab (CD3×B7-H3)	NCT02628535	/	Mesothelioma, Bladder Cancer, Melanoma, Squamous Cell Carcinoma of the Head and Neck, Non-Small Cell Lung Cancer, Clear Cell Renal Cell Carcinoma, Ovarian Cancer, Thyroid Cancer, Breast Cancer, Pancreatic Cancer, Prostate Cancer, Colon Cancer, Soft Tissue Sarcoma	67	I	Terminated (Business decision (not for safety reasons))
		NCT03406949	Retifanlimab (formerly MGA012)	Advanced Solid Tumors	25	I	Active, not recruiting
	ATOR-1015 (CTLA-4 × OX40)	NCT03782467	/	Solid Tumor Neoplasms	53	I	Recruiting
	PRS-343 (HER2 × 4-1BB)	NCT03330561	/	HER2-positive Breast Cancer, HER2-positive Gastric Cancer, HER2-positive Bladder Cancer, HER2-positive Solid Tumor	110	I	Suspended (Partial clinical hold)
		NCT03650348	Atezolizumab	HER2-positive Breast Cancer, HER2-positive Gastric Cancer, HER2-positive Bladder Cancer, HER2-positive Solid Tumor	45	I	Suspended (Partial clinical hold)
**Fusion Protein**	ALT-801 (anti-p53-scTCR×IL2)	NCT01326871	Cisplatin/Gemcitabine	Transitional Cell Carcinoma of Bladder, Urethra Cancer, Ureter Cancer, Malignant Tumor of Renal Pelvis	90	I/II	Unknown
		NCT01625260	Gemcitabine	non-muscle-invasive Bladder Cancer	52	I/II	Unknown
	ALT-803 (IL15-N72D:IL15Rα-Fc)	NCT02138734	BCG	non-muscle-invasive Bladder Cancer	596	I/II	Recruiting
		NCT03022825	BCG	Bladder Cancer	183	II	Recruiting
		NCT03228667	ALT-803 +Pembrolizumab,	Non-Small Cell Lung Cancer, Small Cell Lung Cancer, Urothelial Carcinoma, Head and Neck Squamous Cell Carcinoma, Merkel Cell Carcinoma, Melanoma, Renal Cell Carcinoma, Gastric Cancer, Cervical Cancer, Hepatocellular Carcinoma, Microsatellite Instability, Mismatch Repair Deficiency, Colorectal Cancer	636	II	Recruiting
			ALT-803 + Nivolumab,				
			ALT-803 + Atezolizumab,				
			ALT-803 + Avelumab,				
			ALT-803 + Durvalumab,				
			ALT-803 + Pembrolizumab + PD-L1 t-haNK,				
			ALT-803 + Nivolumab + PD-L1 t-haNK,				
			ALT-803 + Atezolizumab + PD-L1 t-haNK,				
			ALT-803 + Avelumab + PD-L1 t-haNK,				
			ALT-803 + Durvalumab + PD-L1 t-haNK				
**ADC**	Disitamab Vedotin (HER2)	NCT03809013	/	Urothelial Carcinoma	60	II	Recruiting
		NCT04073602	/	Urothelial Carcinoma	18	II	Recruiting
	Enfortumab Vedotin (Nectin-4)	NCT03219333	/	Carcinoma, Transitional Cell, Urinary Bladder Neoplasms, Urologic Neoplasms, Renal Pelvis Neoplasms, Urothelial Cancer, Ureteral Neoplasms, Urethral Neoplasms	219	II	Active, not recruiting
		NCT01409135	/	Tumors, Medical Oncology, Neoplasms	34	I	Completed
		NCT03474107	docetaxel, vinflunine, paclitaxel	Ureteral Cancer, Urothelial Cancer, Bladder Cancer	608	III	Active, not recruiting
		NCT03288545	Pembrolizumab, cisplatin, carboplatin, gemcitabine	Carcinoma, Transitional Cell, Urinary Bladder Neoplasms, Urologic Neoplasms, Renal Pelvis Neoplasms, Urothelial Cancer, Ureteral Neoplasms, Urethral Neoplasms	407	I/II	Recruiting
		NCT03869190	Atezolizumab, Niraparib, Hu5F9-G4, Tiragolumab, Sacituzumab Govitecan, Tocilizumab, RO7122290, RO7121661	Urothelial Carcinoma	385	I/II	Recruiting
	ado-Trastuzumab Emtansine (HER2)	NCT02999672	/	Bladder Cancer, Pancreas Cancer, Cholangiocellular Carcinoma	20	II	Completed
		NCT02675829	/	Solid Tumor Cancers, Lung Cancer, Bladder Cancer, Urinary Tract Cancers	100	II	Active, not recruiting
	Trastuzumab Deruxtecan (HER2)	NCT03523572	Nivolumab	Breast Cancer, Urothelial Carcinoma	99	I	Recruiting
	Sacituzumab Govitecan (Trop2)	NCT03547973	Pembrolizumab	Urothelial Carcinoma	201	II	Recruiting
		NCT03992131	Rucaparib, Lucitanib	Ovarian Cancer, Triple-negative Breast Cancer, Urothelial Carcinoma, Solid Tumor	329	I/II	Recruiting
	Sirtratumab Vedotin (SLITRK6)	NCT01963052	/	Metastatic Urothelial Cancer	93	I	Completed
**Immunotoxin**	Oportuzumab Monatox (anti-EpCAM-scFv×ETA)	NCT02449239	/	Bladder Cancer	134	III	Active, not recruiting
		NCT00462488	/	Urinary Bladder Cancer, Bladder Cancer, Bladder Neoplasms, Bladder Tumors	46	II	Completed
		NCT03258593	Durvalumab	Urinary Bladder Neoplasms	40	I	Recruiting
**mRNA**	mRNA-2752 (encodes IL23, OX40L, IL-36γ)	NCT03739931	Durvalumab	Dose Escalation: Relapsed/Refractory Solid Tumor Malignancies or Lymphoma	126	I	Recruiting
				Dose Expansion: Triple Negative Breast Cancer, Head and Neck Squamous Cell Carcinoma, Non-Hodgkin Lymphoma, and Urothelial Cancer			
**CAR-T cell therapy**	4SCAR-FRα/4SCAR-PSMA	NCT03185468	/	Bladder Cancer, Urothelial Carcinoma Bladder	20	I/II	Recruiting
**Gene therapy**	CG0070	NCT00109655	/	Carcinoma, Transitional Cell, Bladder Neoplasms	75	I	Unknown
		NCT02365818	/	Bladder Cancer	66	II	Completed
		NCT04387461	Pembrolizumab, n-dodecyl-B-D-maltoside	non-muscle-invasive Bladder Cancer	37	II	Not yet recruiting
		NCT04452591	n-dodecyl-B-D-maltoside	Non Muscular Invasive Bladder Cancer	110	III	Not yet recruiting
	rAd-IFN/Syn-3	NCT01162785	/	Bladder Cancer	7	I	Completed
		NCT01687244	/	Superficial Bladder Cancer	40	II	Completed
		NCT02773849	/	Superficial Bladder Cancer	157	III	Active, not recruiting

## Monoclonal Antibodies

With over 90 mAbs approved for a diverse set of diseases like cancer, infectious and autoimmune diseases, monoclonal antibodies are essential in modern medical therapy options (30). While some rely on Fc-mediated effector functions like antibody-dependent cellular cytotoxicity (ADCC) and complement-dependent cytotoxicity (CDC), others achieve clinical benefit through blocking the interaction between the tumor cell and the immune system, like checkpoint inhibitors.

### Checkpoint Inhibitors

Over the last years, immune checkpoint inhibition showed a massive impact on cancer therapies. Particularly antibodies blocking the interaction of the programmed cell death protein 1 (PD-1) and its ligand (PD-L1) showed impressive outcomes in clinical applications (31), resulting in the approval of six anti-PD-(L)1 antibodies (Pembrolizumab, Nivolumab, Cemiplimab, Durvalumab, Avelumab, Atezolizumab). Today, except Cemiplimab, all these anti-PD-(L)1 antibodies are in clinical use for UCs (32, 33).

Studies investigating the expression of PD-1 and PD-L1 in UC samples showed an elevated expression level of PD-(L)1 in high-risk tumors compared to low-risk tumors (34). Immunohistochemistry (IHC) studies on surgically resected urothelial cancer specimens found PD-L1 expression in 21.1% of cases (35). In patients failing BCG-treatment, PD-L1 expression correlated with higher tumor grades. While 7% of pTa tumors expressed PD-L1, 30% of pT3/4 and 45% of CIS tumors exhibited PD-L1 expression. The frequent observation that BCG treatment strongly induces PD-L1 expression might be a reason for the lost effectiveness of BCG treatment over time (36). Furthermore, PD-L1 expression was associated with tumor infiltration of mononuclear cells (36). CD4+ and CD8+ tumor-infiltrating lymphocytes (TILs) were analyzed by flow cytometry for the expression of PD-1. In most patients, PD-1 expression on TILs was highly upregulated compared to peripheral blood lymphocytes (35). While in one study no correlation in PD-(L)1 expression on overall survival was observed (34), another study found that PD-L1 is a significant prognostic factor for postoperative recurrence and survival (35).

After receiving breakthrough therapy designation, priority review status, and accelerated approval, the anti-PD-L1 mAb Atezolizumab (Tecentriq) was the first approved checkpoint inhibitor for MIBC in 2016 (37). In the following year, the Food and Drug Administration (FDA) granted approval for the PD-1 specific mAbs Nivolumab (Opdivo) (37) and Pembrolizumab (Keytruda) (38) as well as for the PD-L1 targeting antibodies Avelumab (Bavencio) (37, 38) and Durvalumab (Imfinzi) (38, 39) for locally advanced or metastatic UC showing tumor progression during or after adjuvant therapy utilizing platinum-containing chemotherapy or neoadjuvant treatment. Today, PD-1/PD-L1 specific antibodies became the standard of care as a second-line treatment for bladder cancer patients.

At the beginning of 2020, Pembrolizumab received approval for the treatment of BCG-unresponsive NMIBC patients unwilling or unfit for cystectomy with CIS with or without papillary tumors after being granted priority review. Today, Pembrolizumab is the only antibody approved for the treatment of NMIBC.

**Figure 2:**
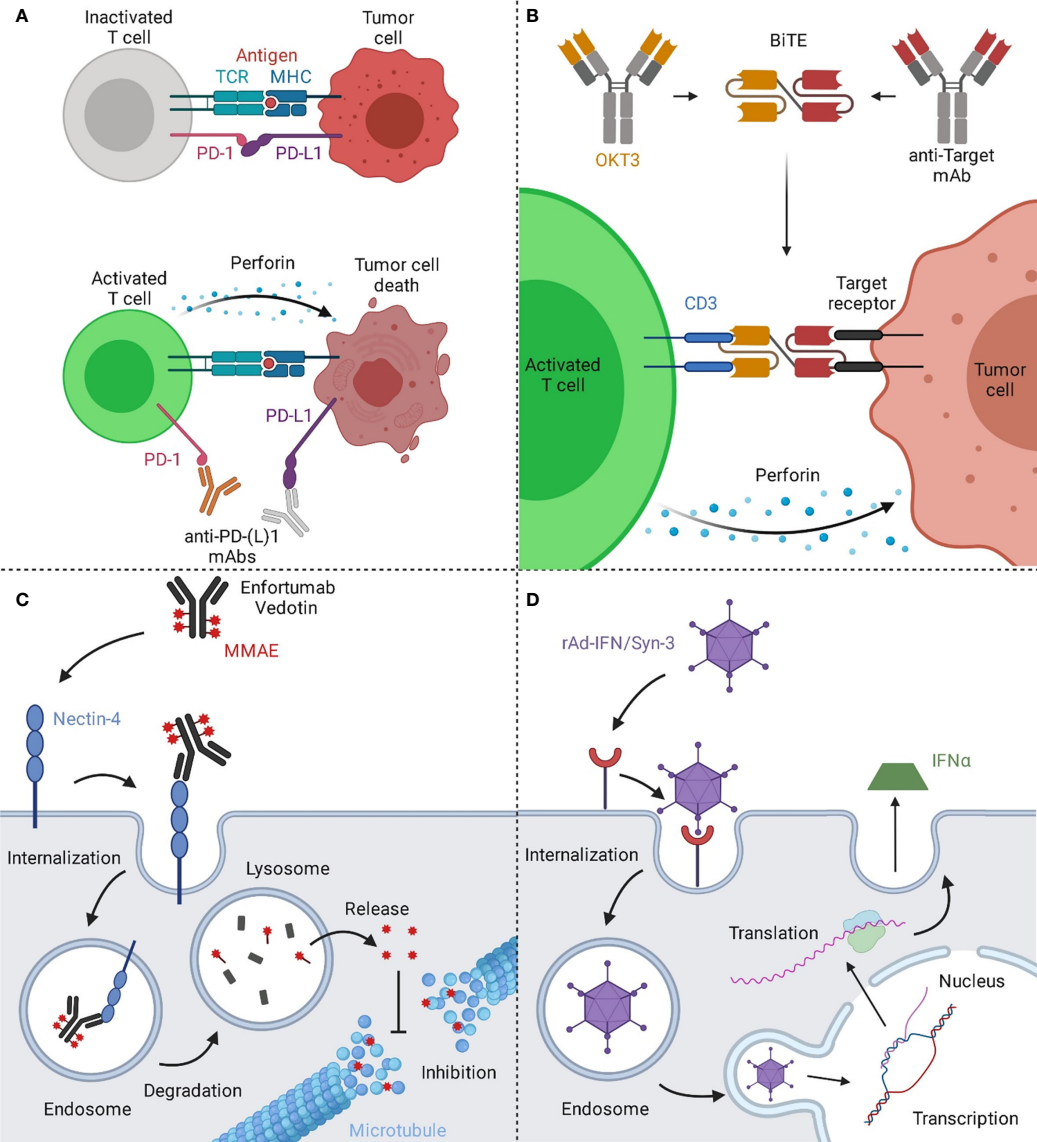
Mechanisms of action of immune and viral therapies for bladder cancer. **(A)** T cells can recognize cancer cells *via* the interaction of their T cell receptor (TCR) with the major histocompatibility complex (MHC) on the target cell, resulting in cytotoxic activity eventually leading to cell death of the malignant cells. However, a prominent escape mechanism of cancer is the upregulation of checkpoint inhibitors like PD-L1, inhibiting cancer-specific T cells from attacking the malignant cells. By blocking the interaction of PD-1 on T cells with its ligand PD-L1 on the tumor cell, T cells are reactivated and can effectively target the tumor. **(B)** Bispecific T cell engagers (BiTEs) consist of two binding arms, simultaneously binding to a tumor-associated antigen on the tumor cell and CD3, a part of the TCR complex, on T cells, generating a synthetic immunological synapse. This results in T cell activation, eventually leading to the induction of a cytotoxic effect against the targeted tumor cell. **(C)** Upon binding of Enfortumab Vedotin to nectin-4, the antibody-drug-conjugate (ADC) gets subsequently internalized into the endosome. Next, the complex is transported to the lysosome, where the ADC is degraded. The valine-citrulline linker is cleaved by cathepsin B resulting in the release of monomethyl auristatin E (MMAE) and the subsequent inhibition of the microtubule, eventually leading to cell death. **(D)** The rAd-IFN/Syn-3 adenovirus is internalized into cells of the bladder after prior instillation. The genetic information of the virus is translocated into the nucleus and upon transduction, these cells produce and secrete IFNα, resulting in an anti-tumor response by activating nearby immune cells. Created with BioRender.com.

While Atezolizumab, Avelumab, and Durvalumab are IgG1 antibodies, Nivolumab and Pembrolizumab belong to the IgG4 isotype (37, 38). Given the number of approved PD-1/PD-L1 specific mAbs for UCs and their impact in other oncologic areas, checkpoint inhibitors have become an essential tool for the treatment of bladder cancer. Their mechanism of action is depicted in **Figure 2A**.

### Lirilumab

Human natural killer (NK) cell activity is modulated by killer cell immunoglobulin-like receptors (KIR) (40). KIRs are classified as activating and inhibitory (41) and recognize predominantly HLA class I as their ligand (42). Blockage of the inhibitory KIR2DL1 has been shown to promote ADCC in tumor models (43).

In 2009, Romagné et al. immunized transgenic mice (44) with BW5417 thymoma cells stably transfected with KIR2DL1, with subsequent booster immunizations utilizing soluble KIR2DL3 (45). Screening of the resulting antibodies resulted in the isolation of the IgG4 antibody IPH2101 (1-7F9), which effectively antagonized KIR signaling by blocking the interaction of HLA class I to inhibitory KIR2DLs. IPH2101 (1-7F9) showed enhanced NK-cell mediated cytotoxicity *in vitro* and *in vivo* (45). An antibody variant with stabilized hinge region termed IPH2102 (Lirilumab) is currently in a phase I study aimed at evaluating its effect in combination with Nivolumab in bladder cancer patients (NCT03532451).

### KMP1

In 2018, Chen and coworkers generated murine monoclonal antibodies by immunizing mice with the bladder cancer cell line EJ. Subsequently, hybridomas were generated, and resulting antibodies were verified for binding to the tumor cells, resulting in the isolation of KMP1. By affinity chromatography and mass spectrometry, CD44 was identified as the antigen of KMP1. IHC studies with patient-derived biopsies showed that patients exhibiting the epitope recognized by KMP1 had a worse prognosis compared to those with a weak KMP1-staining. Furthermore, KMP1 mediated inhibition of proliferation, migration, and adhesion in EJ cells *in vitro* and inhibited tumor growth of EJ-derived tumors in xenografts (46).

CD44 emerged as a promising bladder cancer target as its expression correlates with a higher aggressiveness of the tumor defined by a higher invasion ability compared to CD44 negative cells *in vitro*. Interestingly, it was shown that Interleukin-6 (IL-6) signaling facilitates a favorable microenvironment for CD44 expression (47). Furthermore, CD44 overexpressing tumors exhibited a lower complete response rate and lower survival. Interestingly, *in vivo* experiments showed that CD44+ tumors were more resistant to irradiation, both for immunocompromised and immunocompetent hosts (48).

### Vofatamab

Vofatamab is a clinical-stage mAb targeting the fibroblast growth factor receptor 3 (FGFR3). FGFR3 signaling has been linked to development, differentiation, growth, and survival (49). In patients suffering from bladder cancer, 13 different activating missense mutations have been identified, with three alternations, R248C, S249C, and Y375C, accounting for over 85% of all observed mutations (50). The cysteine residues generated by the mutations presumably lead to ligand-independent receptor dimerization and subsequent activation (51). Furthermore, FGFR3-fusion proteins were observed, where the complete FGFR3 sequence except the final exon was C-terminally fused with the sequences of TACC3 (52, 53) or BAIAP2L1 (54), resulting in a phenotype associated with higher grades (50, 55).

One study on FGFR3 in the context of NMIBC found that 32% of T1 tumors exhibit FGFR3 mutation variants, and 96% of those are overexpressed. In tumors exhibiting a wild-type FGFR3 gene (68%), overexpression was found in 47% of cases. Overall, 63% of the study population exhibited overexpression of FGFR3 (56). Consistently, another study showed that 85% of tumors exhibiting mutations within FGFR3 showed overexpression while being low-grade. Furthermore, it seems that higher FGFR3 expression is elevated in more differentiated tumors (57). This is in line with the finding that higher FGFR3 expression was found in pTa stage tumors compared to pT1 tumors and is therefore associated with lower-grade (57, 58) as well as a favorable prognosis. In contrast, wtFGFR3 expression did not show any influence on clinical parameters (56). Only 42% of tumors exhibiting wtFGFR3 showed overexpression, where 66% were high-grade, and 68% invasive (58).

In 2009, Qing and coworkers isolated an anti-FGFR3 monoclonal antibody, termed R3mab *via* phage display, which blocked the ligand binding as well as receptor dimerization (59). Furthermore, R3mab was able to bind not only wtFGFR3 but also the most common FGFR3 mutants and inhibited proliferation as well as FGFR3 signaling in bladder cancer cells, exhibiting wild type and mutated variants of FGFR3. In subsequent *in vivo* studies utilizing xenografts of either WT or mutated FGFR3 cells, R3mab revealed remarkable anti-tumor effects. In 2015 R3mab, now termed as Vofatamab, was tested in a phase I/II dose-escalation study alone or in combination with docetaxel for metastatic urothelial cell carcinoma (NCT02401542). 20% of enrolled patients experienced serious adverse events when treated with Vofatamab as a monotherapy and over 50% when treated in combination with docetaxel, resulting in the termination of the study. Furthermore, a phase I study in 2016 combined with Pembrolizumab (NCT02925533) was conducted, but terminated as well due to safety concerns. A phase I/II study in 2017 for locally advanced or metastatic urothelial cell carcinoma (NCT03123055) was terminated since over 46% of patients experienced serious adverse events.

## Bispecific Antibodies

Over recent years, bispecific antibodies (bsAb) have gained interest of pharmaceutical and academic research. The dual targeting binding properties can mediate mechanisms of action, being impossible to accomplish with their monospecific counterparts or a combination of multiple standard IgGs (60). To this day, only three bispecific antibodies were approved. One is the by now withdrawn Catumaxomab for the treatment of ascites, the second one is Blinatumomab, which is approved for the treatment of acute lymphatic leukemia (ALL), and Emicizumab, approved for hemophilia A treatment, is the third. However, a variety of bsAbs is in (pre-)clinical development for the treatment of different indications, including bladder cancer, exhibiting a diverse spectrum of antibody formats.

### Anti-CD3 Bispecifics

**Figure 3:**
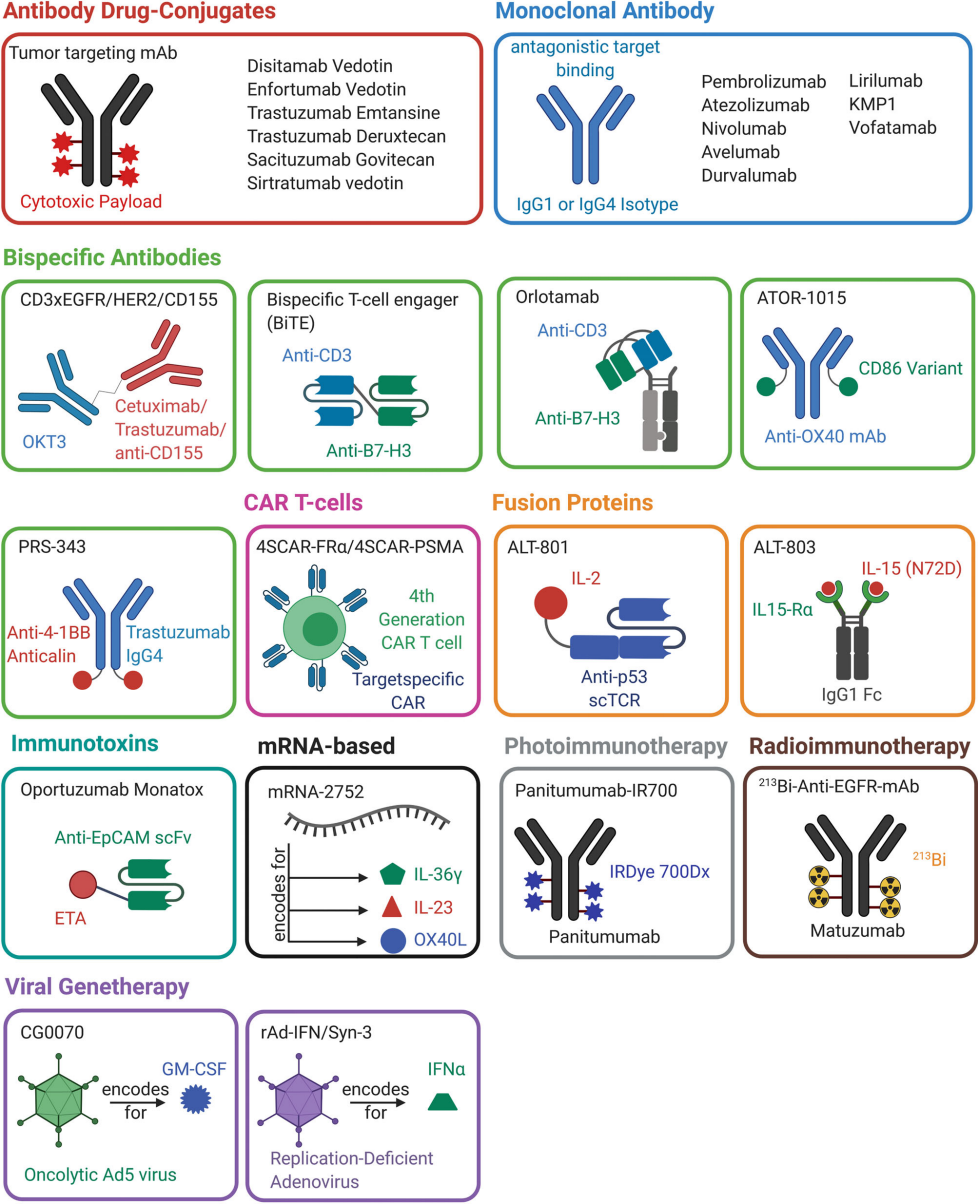
Overview of antibody-, protein-, mRNA-, cell- and viral-based drugs that are approved or are in (pre-)clinical development for the treatment of bladder cancer. The molecules belonging to one drug class are marked in the same color. A schematic representation of the molecular architecture is given. Created with BioRender.com.

T-cell engagers are bispecific molecules that bridge cytotoxic T cells to tumor cells leading to the generation of a synthetic immunologic synapse. This results in cytotoxic activity on tumor cells mediated by T-cells. Commonly, CD3-specific antibodies are utilized as T cell binding moieties (61). The mechanism of action of T cell engagers is depicted in **Figure 2B**. The molecular structure T cell engagers, as well as of all other discussed molecules, is illustrated in **Figure 3**.

In 2018 Juan Ma and coworkers generated CD3×EGFR and CD3×HER2 bispecific antibodies by reacting the anti-epidermal growth factor receptor (EGFR) mAb Cetuximab or the anti-HER2 mAb Trastuzumab, respectively, with sulfo-succinimidyl 4-(N-maleimidomethyl)cyclohexane-1-carboxylate (sulfo-SMCC), followed by conjugation with OKT3 after activation with Traut’s reagent (62). The mouse-derived OKT3 mAb recognizes the CD3ϵ domain in the T cell receptor (TCR) complex and is commonly used for the construction of T cell engagers, as e.g., Blinatumomab (63). By analyzing human-derived active T cells, it could be demonstrated that the bispecific constructs exhibited a stronger cytotoxic activity against bladder cancer cells and mediated secretion of the activation markers Interferon gamma (IFN-γ), tumor necrosis factor alpha (TNF-α), and IL-2 (62). The following year, the same group utilized this system to generate a CD3×CD155 bispecific antibody (64).

CD155, also known as nectin-like protein 5, belongs to the Ig superfamily and is overexpressed in multiple malignancies, including lung adenocarcinoma, pancreatic, colon cancer as well as in muscle-invasive bladder cancer (65–70).

Peripheral blood mononuclear cells (PBMC), isolated from healthy donors and bladder cancer patients, were activated and subsequently treated with the CD3×CD155 bsAb, resulting in cytotoxic effects against bladder cancer cell lines (64). A major drawback in chemical antibody conjugation strategy lies in the fact that it is not site-specific, and therefore it yields a heterogenous population of bispecific molecules.

Several alternative routes exist to overcome this problem. A frequently used format is based on the genetic fusion of two single chain Fv (scFv) fragments that consist of an antibody variable domain of the heavy and the light chain connected by a linker sequence, each addressing different targets resulting in a bispecific T cell engager (BiTE)-like architecture (**Figure 3**) (71). In frame of development of bispecific T cell engagers for the treatment of bladder cancer, Li and coworkers generated a tandem BiTE-based CD3×B7-H3 bispecific antibody (72, 73).

B7-H3 is naturally expressed by antigen-presenting cells (APC) and is involved in inhibition of T cells (74–76). However, overexpression can also be found on several cancer cells (77–83), where B7-H3 plays a role in cell migration and invasion (84). Interestingly, B7-H3 exhibits a high expression level in bladder cancer cells. *In vitro* cytotoxicity studies of this CD3×B7-H3 BiTE, as well as *in vivo* experiments, revealed a notable cell-killing activity of bladder cancer cells, which was further improved by combination with trametinib, a small molecule MEK inhibitor (72).

The only T cell engaging molecule that was in clinical testing for bladder cancer was Orlotamab/MGD009 (NCT02628535), a humanized Dual Affinity Re-Targeting (DART) protein which comprises a CD3 and a B7-H3 binding moiety (85). DART molecules exhibit a diabody-like architecture with the VH domain of one binder linked to the VL of the second binder and the VH of the second binding moiety attached to the VL of the first. Furthermore, disulfides are utilized to stabilize the construct (60, 86). The DART of MGD009 is linked to an Fc fragment, resulting in a monovalent antibody-like molecule (**Figure 3**). The Fc part is mutated to reduce effector functions while allowing recyclization *via* the neonatal Fc receptor to mediate extended *in vivo* half-life (85). The first clinical testing of Orlotamab/MGD009 against bladder cancer, among others, was terminated due to business decisions and not for safety issues (85) (NCT02628535). However, a phase I study is currently ongoing for the treatment of advanced solid tumors (87) (NCT03406949).

### ATOR-1015

Immune checkpoint inhibitors such as the anti-CTLA-4 mAb Ipilimumab can have significant effects in cancer patients. Kvarnhammar and coworkers developed a bispecific CTLA-4×OX40 bsAb (ATOR-1015) (88). To this end, OX40 binding Fab domains were isolated from an scFv phage display library. For CTLA-4 binding, the V-like domain of the CTLA-4 ligand CD86 was engineered in five residues to achieve a 100-fold increased affinity towards CTLA-4. This engineered CD86 domain was C-terminally fused to the kappa light chain of the anti-OX40 IgG1 molecule (88) (**Figure 3**). The resulting bispecific antibody bound CTLA-4 with 3.0 nM affinity and blocked the interaction with CD80 and CD86.

The co-stimulatory molecule OX40 (CD134) belongs to the next generation of immune therapeutic targets. CTLA-4 and OX40 are upregulated in tumor-infiltrating, but not peripheral, regulatory T cells (Tregs). ATOR-1015 binds domain 2 on OX40 with an affinity of 1.6 nM and blocks interaction with OX40L and furthermore induced T cell activation *in vitro* by blocking CTLA-4. By crosslinking OX40 upon bind to either CTLA-4 or to FcγRIIIa (CD16a), ATOR-1015 leads to NK-cell or T cell-mediated depletion of OX40-overexpressing Tregs. Those Tregs can negatively regulate the immune response (89–92), and their depletion can enhance anti-tumor activity (93–95). By targeting both receptors in a bispecific manner, the activity of the bsAb is not only directed to the tumor side, eventually introducing anti-tumor responses, but also may result in a more favorable safety profile and better efficacy compared to standard anti-CTLA4 mAbs (88, 96).

*In vivo* experiments utilizing the MB49 bladder cancer model showed a reduction in tumor growth and prolonged survival. Furthermore, ATOR-1015 enhanced the effect of anti-PD1 treatment in *in vivo* models. As ATOR-1015 shows promising results in preclinical cancer models, including colon, pancreas, and bladder cancer, it is currently in phase I clinical trials (NCT03782467).

### PRS-343

PRS-343 is a bispecific fusion protein targeting HER2 and 4-1BB (CD137), which is a costimulatory receptor on T cells. Despite 4-1BB being a therapeutic target of major interest, prior clinical studies utilizing the monospecific 4-1BB-binding IgG4 antibody Urelumab were stopped due to hepatoxicity (97). The bispecific antibody PRS-343 has the potential to overcome these side-effects by its tumor-specific mechanism of action. Upon accumulation in HER2-positive tumors and clustering of HER2, 4-1BB is clustered on nearby T cells, resulting in T cell activation and a cytotoxic response against the tumor.

The PRS-343 architecture is based on a Trastuzumab variant and a 4-1BB specific anticalin (98) (**Figure 3**). Anticalins are engineered variants of tear lipocalin and neutrophil -gelatinase–associated lipocalin (NGAL), where multiple loops are randomized by concerted mutagenesis (98–100). Via phage display, a 4-1BB-binding anticalin, termed J10, was isolated from an anticalin library. This molecule exhibits a 2 nM affinity, shows cross-specific binding to 4-1BB from cynomolgus monkeys, and does not block ligand binding (98). Trastuzumab VH and VL domains were subcloned into an IgG4 format to silence Fc-mediated effector functions. Furthermore, the S228P mutation was implemented, suppressing the naturally occurring Fab-arm exchange found in IgG4 molecules (101). Fab-arm exchange describes the naturally occurring and dynamic dissociation of an IgG4 homo-tetramer into two half-molecules consisting of one heavy and one light chain. This is followed by the reassociation with a second half antibody, leading to an antibody with two different Fab arms (102). This mechanism would lead to antibodies with reduced specificity and therapeutic efficacy and is therefore avoided in PRS-343 (103). Additionally, the F234A and the L235A mutation, which reduce the binding to Fcγ-receptors and further silence effector function, were implemented in PRS-343 (104, 105). Since the distance between effector and target cells is crucial for T cell activation and cytotoxicity (105), the anticalin moiety was N- and C-terminally fused to the heavy and light chains of the engineered Trastuzumab variant utilizing a (G_4_S)_3_ linker. While all variants exhibited similar biophysical properties, the C-terminal heavy chain fusion showed the strongest T cell activation.

*In vivo* experiments demonstrated recruitment of TILs, while lymphocytes in peripheral blood remained unaffected (98). Due to its favorable properties, phase I dose-escalation studies were conducted in patients with HER2-positive cancers, including bladder cancer, either as a monotherapy (NCT03330561) or in combination with Atezolizumab (NCT03650348). Both studies were suspended, but very recently, the clinical hold was lifted by the FDA.

## Fusion Proteins

Like bsAbs, fusion proteins can mediate mechanisms of action that are impossible to achieve with a mixture of the parental, monofunctional fusion partners. For example, Etanercept or Belatacept are antibody-derived Fc fusion proteins where the Fab fragment of the antibody is replaced by either the extracellular domain of tumor necrosis factor receptor or CTLA4, respectively. While those molecules are approved for the treatment of rheumatoid arthritis or organ transplant rejection for years (106–108), no fusion proteins are approved for bladder cancer yet, but some have entered clinical development.

### ALT-801

In 1995, Theobald and coworkers used transgenic mice to generate cytotoxic T lymphocytes (CTL) targeting the human p53 protein (109). From the population of CTLs the clone no. 5, specific to the p53 peptide 264–272, was isolated by limiting dilution (110). To generate a soluble T-cell receptor, Belmont et al. amplified the Vα and Vβ/Cβ regions by RT-PCR. Subsequently, the C-terminal end of the Vα was fused to the N-terminal end of the Vβ utilizing a (G_4_S)_4_ linker. The Cβ region directly fused to Vβ was truncated to lack the final cysteine as well as the transmembrane and cytoplasmic regions. Via a peptide linker (encoding for VNAKTTAPSVYPLAPV), human IL-2 was linked to the single-chain TCR (scTCR) (**Figure 3**). The construct was produced in mammalian cell culture and the resulting fusion protein bound to p53-peptide-loaded MHC on T2 cells, as well as to the IL-2 receptor. Furthermore, the scTCR-IL-2 construct exhibited similar biologic effects *in vitro* as recombinant IL-2. It was shown that this construct could bring p53-peptide-loaded MHC-bearing cells in proximity with cells exhibiting the IL-2R. Pharmacokinetic studies showed a half-life in mice of 1.6 to 3h, which is elevated compared to free IL-2, as it has a half-life of 5 min. *In vivo* experiments using xenografts showed beneficial outcomes of the scTCR-IL-2 construct in comparison to IL-2 alone (111). To reduce the immunogenicity of the construct, the murine Cβ was exchanged to its human counterpart while retaining a promising profile in *in vivo* experiments (112). In 2011 and 2012, phase I/II studies of NMIBC and bladder cancer were initiated, but the current status is unknown (NCT01326871, NCT01625260).

However, this engineering approach’s versatility was demonstrated as Zhu and coworkers substituted the IL-2 moiety with an IL-15 variant (N72D), which exhibits an improved binding towards the IL-15Rβ, resulting in an scTCR fusion construct exhibiting a super agonistic mechanism of action for the IL-15 pathway (113).

### ALT-803

Based on the IL-15 N72D mutein, Han and coworkers constructed in 2011 a fusion protein of the sushi domain of IL-15Rα (aa 1-66) and a C-terminal human IgG1 Fc part. Subsequently, plasmids encoding for IL-15 N72D and IL-15Rα-hFc were co-transfected in Chinese hamster ovary (CHO) cells. IL-15 is poorly produced in mammalian cell culture due to its fast degradation. However, in complex with IL-15Rα, the cytokine is shielded from degradation in the endoplasmic reticulum, resulting in high expression levels of the IL-15 N72D:IL-15Rα-hFc complex. Utilizing ion-exchange chromatography methods allowed for the specific purification of IL-15Rα-hFc molecules fully occupied by two IL-15 N72D variants (114) (**Figure 3**).

IL-15 stimulates the proliferation of NK cells and T cells and induces the generation of CD8+ cytotoxic lymphocytes. Naturally, both IL-15 and IL-15Rα are expressed on dendritic cells and interact with NK cells and T cells in trans, which express cell-bound IL-2/IL-15Rβ and the common gamma chain (γc), resulting in heterotrimeric receptor formed inside the immunologic synapse (115). ALT-803 can bind to IL-2/-15Rβ and γc on T cells and NK cells and induce the IL-15 pathway leading to stimulation of cell proliferation.

Pharmacokinetic experiments demonstrated that the complex exhibited an *in vivo* half-life of 18 to 25 h, which is significantly enhanced compared to wtIL-15 (0.64 h). *In vivo*, the complex mediated proliferation of NK cells and CD8+ T cells in mice (114). As this super agonistic construct can activate NK cells, it was *in vitro* demonstrated that the ALT-803 is able to modulate ADCC activity of a cancer-specific antibody. Furthermore, it led to the upregulation of activating NK cell receptors, antiapoptotic factors, and factors involved in the NK cytotoxicity while simultaneously downregulating NK cell inhibiting factors (116). Furuya and coworkers investigated the effect of ALT-803 in an orthotopic mouse model, demonstrating that subcutaneous administration of ALT-803 is non-inferior to intravesical treatment of BCG. While subcutaneous administered ALT-803 activated CTLs, NK cells and NKT cells, in combination with intravesical BCG additional immunomodulating cytokines in the serum were detected (117). Additional mouse models demonstrated that ALT-803 increased PD-L1 expression in tumors *in vivo* and elucidated the safety and efficacy of combination treatment with PD-L1-specific checkpoint blockage, paving the way for clinical combination treatment (118). Currently, ALT-803 is in phase I/II and phase II studies investigating its effect on patients suffering from NMIBC or bladder cancer, respectively, as monotherapy or in combination with BCG (NCT02138734, NCT03022825). Furthermore, a currently recruiting phase II study will investigate the efficacy of ALT-803 in combination with the checkpoint inhibitors Pembrolizumab, Nivolumab, Atezolizumab, Avelumab or Durvalumab. One cohort, in addition to the combination of ALT-803 with one of the checkpoint inhibitors, will be weekly dosed with PD-L1 t-haNK cells (NCT03228667). These cells are derived from NK-92 cells, exhibit the high-affinity CD16 receptor, produce an IL-2 variant, which is retained in the endoplasmic reticulum, and carry an additional chimeric antigen receptor addressing PD-L1 (119).

## Antibody-Drug-Conjugates

**Figure 4:**
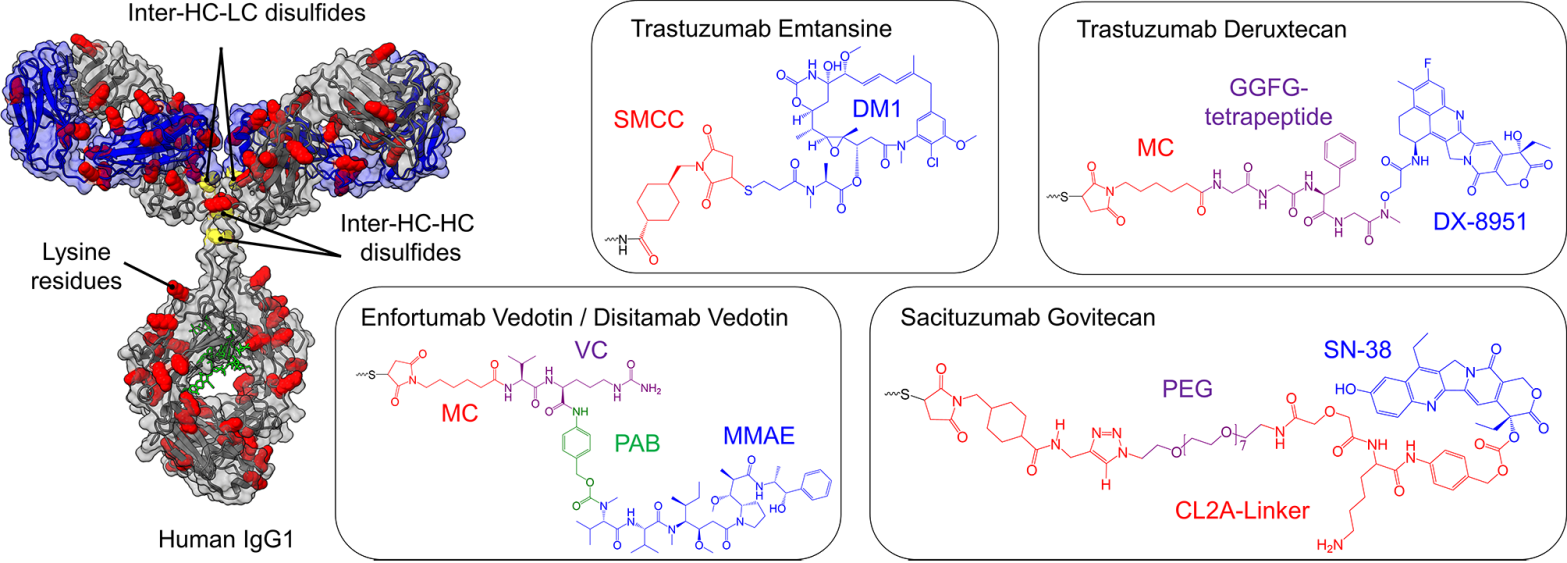
Cytotoxic payloads and conjugation strategies of approved and clinical-stage antibody-drug-conjugates (ADCs) for the treatment of bladder cancer. On the left, a human IgG1 antibody is shown, where cysteine (yellow) and lysine residues (red) are highlighted, the glycosylation is marked in green, while the light chains are depicted in blue. The heavy chain is illustrated in grey. Depending on the conjugation strategy, either cysteine residues or lysine residues are modified with the toxins illustrated on the right side. Except for ado-Trastuzumab Emtansine, all clinical-stage ADCs for bladder cancer are based on the partial reduction of inter-chain disulfide bonds (yellow), resulting in a maximal drug to antibody ratio (DAR) of 8, while simultaneously reducing the number of potential ADC variants. In ado-Trastuzumab Emtansine conjugation is achieved by coupling to lysine residues (red). The respective linkers and toxins of the payloads are color-coded and named separately. SMCC, succinimidyl 4-(N-maleimidomethyl)cyclohexane-1-carboxylate; DM1, maytansine; MC, maleimidocaproyl; PAB, p-aminobenzyl; MMAE, monomethyl auristatin E; PEG, polyethylene glycol.

Antibody-drug-conjugates (ADC) comprise a cancer-targeting monoclonal antibody, which is conjugated to a cytotoxic agent (**Figure 3**). The specificity of the mAb ensures a selective targeting of tumor cells. Upon antigen-binding, ADCs are internalized by receptor-mediated endocytosis and trafficked to the endosome and ultimately to the lysosome. Depending on the linker utilized, acidic pH or lysosomal proteases mediate linker cleavage, resulting in the release of the toxin and eventually cell death (120, 121). The linkers, the toxins, and the conjugation sites of all clinical-stage ADCs for bladder cancer are illustrated in **Figure 4**. While coupling efficiency and heterogeneity of the conjugate are common hurdles (122), nine ADCs are approved for several malignancies, including solid tumors such as breast cancer and hematologic malignancies (123–127). Currently, 16 ADCs are in phase II and III studies, with many more in phase I studies and preclinical development (121). With the recent approval of Enfortumab Vedotin (Padcev) and the clinical evaluation of multiple other ADCs, the focus is shifting to urothelial malignancies (128).

Two marketed ADCs are based on the monoclonal antibody Trastuzumab targeting the human epidermal growth factor receptor 2 (HER2), a well-studied tumor antigen overexpressed in a variety of malignant diseases, including breast cancer (129, 130) and lung cancer (131, 132). Furthermore, HER2 emerged as a promising therapeutic target for bladder cancer:

Fluorescence *in situ* hybridization (FISH) and IHC studies of tissue arrays derived from high- and low-grade UCs, as well as papillary urothelial neoplasms, revealed that up to 9% of high-grade UCs exhibit a HER2 gene amplification, which can be associated with higher recurrence and a worse prognosis. Additionally, a subset of high-grade NMIBC patients exhibits a HER2 gene amplification, which can be correlated with aggressive tumor growth (133). Consistently, a silver-enhanced *in situ* hybridization (SISH) analysis of NMIBC patients showed that 4.2% of the tumors exhibited stronger HER2 staining. All HER2-positive NMIBC samples belonged to the high-grade subset, and HER2-expression is an independent predictor of tumor prognosis (134). Today, multiple anti-HER2-ADCs are in clinical development for the treatment of bladder cancer (128).

### Disitamab Vedotin

In order to generate a novel HER2-specific antibody, Yao and coworkers immunized mice with the extracellular domain (ECD) of HER2, extracted the spleen, and generated hybridomas (135). The clone exhibiting the best binding activity was sequenced and subsequently humanized by CDR grafting. The resulting mAb variant was termed Hertuzumab and showed a higher affinity compared to gold-standard Trastuzumab. By partial reduction of intermolecular disulfide bonds and a subsequent conjugation utilizing a thiol-reactive maleimido-valine-citrulline-dipeptide-linker, monomethyl auristatin E (MMAE) was conjugated, resulting in the ADC RC48 (135). MMAE inhibits tubulin polymerization, resulting in phase arrest and apoptosis (136). The linker is effectively cleaved by human cathepsin B upon internalization in HER2 positive cells but stable in human plasma (137). *In vivo* studies using xenografts demonstrated a significant and durable regression, underlining the high potency of this ADC (135).

In 2015, a dose-escalating phase I study was conducted in HER2-overexpressing cancer patients, showing a manageable safety profile while exhibiting low liver toxicity (137, 138) (NCT02881190). Currently, RC48, renamed as Disitamab Vedotin, is in phase II studies for locally advanced or metastatic urothelial cancer in HER2 overexpressing (NCT03809013) or HER2-negative patients (NCT04073602) and recently gained breakthrough therapy status.

### Enfortumab Vedotin (Padcev)

For the treatment of PD-1/PD-L1 unresponsive patients suffering from locally advanced or metastatic UC, the FDA approved Enfortumab Vedotin in 2018 (139). This was based on a clinical trial including 125 participants (NCT03219333), where 44% of enrolled patients had tumor shrinkage or growth arrest, including 12% showing a complete response, meaning the tumor disappeared entirely (140). This ADC consists of a nectin-4 targeting antibody, conjugated to a microtubule inhibitor and was granted breakthrough therapy designation, priority review status, and accelerated approval.

The cell adhesion molecule (CAM) nectin-4 belongs to the family of nectin-like proteins (Necl), consisting of nectin-1, -2, -3, -4, and -5, which trans-interact with one another (141–143). Besides breast (144), lung (145), and ovarian cancer (146), it is expressed in malignancies of the bladder (147), where it is involved in metastasis and proliferation (145, 148–150).

The parental human IgG1 antibody (AGS-22M6) was generated by utilizing the XenoMouse technology, where transgenic mice are genetically engineered with a humanized humoral immune system (151). Immunization was performed with the extracellular domain (ECD) of human nectin-4, followed by the generation of over 50 hybridomas (152). Besides binding to human nectin-4, AGS-22M6 showed cross-specificity to its monkey and rat orthologues. Using flow cytometry, it was demonstrated that AGS-22M6 bound its human target with a KD value of 10 pM, which was also confirmed for its MMAE-conjugated counterpart. By deletion mutagenesis of nectin-4, it was shown that the epitope of AGS-22M6 is located on the V-domain, enabling inhibition of the heterodimerization with nectin-1 (152). For recombinant expression, VH and VL genes were extracted from the hybridoma and subcloned into expression vectors for production in CHO cells. For ADC generation, the microtubule-disrupting toxin MMAE was conjugated utilizing the cleavable linker maleimidocaproylvaline-citrulline-p- aminobenzyloxycarbonyl (136), which was conjugated by partial reduction of interchain disulfide bond (153, 154), resulting in a drug to antibody ratio (DAR) of approximately 4 (152). In preclinical xenograft models, the ADC showed significant growth inhibition in breast, pancreatic, lung, and bladder cancer models (152), paving the way for its phase I study, which started in 2011 (NCT01409135).

Currently, Enfortumab Vedotin is in further clinical trials investigating its effect on bladder cancer in comparison to chemotherapy (NCT03474107), in combination with checkpoint inhibitors and chemotherapy (NCT03288545), or combined with other immunotherapies after failure of platinum-containing chemotherapy (NCT03869190).

### ado-Trastuzumab Emtansine (Kadcyla)

In 2008, Phillips and coworkers conjugated the HER2-specific antibody Trastuzumab to a maytansine derivative (DM1) *via* the non-reducible thioether linker SMCC (155, 156). Trastuzumab exhibits 88 lysine residues that could potentially be utilized for conjugation. While the DAR is approximately 3.5, at least 70 lysine residues are partially conjugated, resulting in a heterogeneous product (157). The resulting ADC, termed T-DM1, was effective in Trastuzumab-sensitive and -insensitive cancer models *in vitro* as well as *in vivo*. T-DM1 is internalized upon binding to HER2, resulting in accumulation in the endosome (155, 158). As the linker utilized is non-reducible, DM1 release occurs by antibody degradation in the lysosome (156, 159). After lysosomal escape, DM1 inhibits microtubule assembly, resulting in cell death (155, 160). T-DM1, further termed as ado-Trastuzumab Emtansine, was approved in 2013 for HER2 positive breast cancer. Recently, a phase II study investigating the tumor response in HER2 expressing tumors, including bladder cancer, was completed (NCT02999672). In cohort 1, 13 patients with locally advanced or metastatic urothelial bladder carcinoma were dosed with 3.6 mg/kg qw, after the safety of Trastuzumab Emtansine was assessed by an independent data management committee after dosing the first six patients with 2.4 mg/kg qw. Partial response (defined as a 30% decrease in the sum of the diameters of the target lesions taking as a reference the baseline sum diameter according to the RECIST 1.1 criteria) was observed in 38.5% of patients. No patients achieved complete response. Median OS (95%CI) was 7.03 months (3.75 to NE). A second study on HER2 amplified or mutated cancers, including bladder cancer, is currently ongoing (NCT02675829).

### Trastuzumab Deruxtecan (Enhertu)

DS-8201a is another HER2-targeting ADC. It is based on Trastuzumab, which was conjugated with DX-8951 (DXd), a topoisomerase I inhibitor *via* a GGFG peptide linker, using maleimide chemistry, after partial reduction of interchain disulfides. This tetrapeptide linker is cleaved by lysosomal proteases cathepsins B and L in HER2-positive tumor cells upon receptor-mediated endocytosis (161). The coupling results in a high DAR of 7.7, enabling anti-tumor effects even in HER2 low-expressing models not being feasible with ADCs exhibiting lower DAR (including T-DM1/ado-Trastuzumab Emtansine) (161, 162).

This higher DAR might help to overcome drug resistance, which is observed in T-DM1 treated patients. Even though the drug resistance in the context of T-DM1 is not fully understood, one of the main reasons might be the downregulation of HER2, impaired internalization, enhanced recycling of HER2, and lysosomal degradation (155, 163). However, due to its different mechanism of action and efficacy in low HER2-expressing cancers, it is hypothesized that DS-8201a might be effective against T-DM1 resistant tumors (161). Currently, DS8201a, renamed as Trastuzumab Deruxtecan, is under clinical investigation for advanced breast and urothelial cancer in combination with Nivolumab (NCT03523572) (164). Trastuzumab Deruxtecan is approved for treatment of patients suffering from unresectable or metastatic HER2-positive breast cancer.

### Sacituzumab Govitecan (Trodelvy)

In 1990, Stein and coworkers utilized the hybridoma technology to generate the antibody RS7-3G11 by immunizing mice with crude membrane preparations of squamous cell carcinoma of the lung (165). It was observed that the resulting mAb RS7-3G11 is specific to multiple malignancies, including breast, colon, renal, and prostate cancers (166). In 1995, Basu and coworkers identified Trop2 as the target of RS7-3G11 (167).

Trop2, also known as human trophoblastic cell surface antigen 2, is an oncogene involved in tumorigenesis and tumor invasion (168–170). With a low expression in healthy tissue and being upregulated in several cancers, it emerged as a promising target (171, 172).

It was observed that RS7 facilitates fast internalization and was evaluated for *in vivo* efficacy (173). Upon humanization by CDR-grafting, the now termed hRS7 was subjected to radio-labeled *in vivo* studies (174). Additionally, ADCC effects in cervical cancers were observed utilizing hRS7 (174). Later on, hRS7 was conjugated with the topoisomerase inhibitor SN-38, resulting in the ADC IMMU-132 or Sacituzumab Govitecan (175, 176). Conjugation was performed by mild reduction of interchain disulfides followed by formation of a thioether bond, resulting in DARs of less than 4 (176). Subsequent phase I studies showed acceptable toxicity and promising results (177). Currently, Sacituzumab Govitecan is in clinical phase II studies for metastatic UC (NCT03547973) and phase I/II studies in combination with chemotherapy for multiple carcinomas, including UC (NCT03992131). In early 2020, Sacituzumab Govitecan was approved under the name Trodelvy for the treatment of triple-negative breast cancer.

### Sirtratumab Vedotin

Utilizing tumor microarrays, SLIT and NTRK-like protein 6 (SLITRK6) were found to be expressed in 88% of bladder cancer specimens, of which 67% showed strong to moderate expression. Furthermore, transitional cell carcinoma exhibited SLITRK6 expression in 90% of cases and metastatic bladder cancer in 100% of cases (178).

In order to generate an anti-SLITRK6 ADC, the Xenomouse technology was employed by performing immunization with SLITRK6 expressing cells or recombinant SLITRK6 protein, resulting in hybridomas clones. The clone AGS-15C was identified for its binding to SLITRK6 and subcloned for recombinant expression (178). Subsequently, ASG-15C was conjugated to MMAE *via* a valine-citrulline dipeptide linker, resulting in the ADC ASG-15ME. The ADC caused complete growth inhibition in xenograft lung cancer models. Interestingly, approximately 5% of tumor cells were negative for SLITRK6 expression, underlining the effect this ADC has on heterogenic cancers, which are typically found in patients (178). AGS-15ME, also termed Sirtratumab Vedotin, was tested in a phase I clinical study on metastatic urothelial cancer (NCT01963052).

With the recent approval of Enfortumab Vedotin and the large number of additional toxin-coupled mAbs in clinical testing for bladder cancer, ADCs have the chance to gain a profound impact in the treatment of bladder cancer. The mechanism of action of the approved Enfortumab Vedotin is depicted in **Figure 2C**.

## Photoimmunotherapy

The photoimmunotherapeutic approach is based on an ADC-like molecule that carries an inactive cytotoxic agent to the tumor cell by conjugation to a monoclonal antibody (**Figure 3**). By irradiation with light of the appropriate wavelength, the cytotoxic agent is activated and is able to mediate effective tumor-killing (179).

### Panitumumab-IR700

The photoimmuno-conjugate Panitumumab-IR700 is based on the EGFR-specific mAb Panitumumab. RT-PCR analysis of TURB bladder washings of stage Ta, T1, and Tis NMIBC patients revealed that EGFR expression is 1.7-fold elevated in low-risk patients and up to 3-fold higher in high-risk patients (180). Other studies found by IHC that 15.1% of UC patients had low and 26.2% had high EGFR expression (181), where one study found EGFR overexpression in 74% of UC patients (182). No association of EGFR expressing UCs and mutations in the tyrosine kinase domain are known to this date (182). However, EGFR expression was correlated with prognostic factors like grade, deep muscle invasion, and recurrence (180, 181). Other IHC studies of tissue microarrays revealed that 70% of samples exhibited a relative staining intensity of 1 or higher staining. Of those samples, 16% had staining intensities of 3, and 31% had staining intensities of 4 stainings. A higher rate of positive stainings was observed in squamous tumors (94%) in comparison with nonsquamous tumors (69%). Interestingly, no correlation between EGFR staining intensity and T stage was observed (183). However, Kassouf et al. found that tumors simultaneously showing a high EGFR and low HER4 expression display a more invasive phenotype in conjunction with a short recurrence time (184). In consistence, a study investigating the expression of all HER-family members in bladder cancer found that while EGFR and HER2 expression rates alone do not correlate with survival, higher EGFR or HER2 expression combined with low HER3 and HER4 expression result in a worse prognosis (185).

In 2017 Railkar et al. conjugated the EGFR-specific and FDA-approved mAb Panitumumab *via* NHS ester chemistry with IRDye 700Dx, generating Panitumumab-IR700, intending to create a targeted phototherapy, termed photoimmunotherapy (PIT) for bladder cancer (183). IR700 is a hydrophilic agent with low cytotoxicity. Its cytotoxic nature is activated by the association to the cell membrane and subsequent activation using near-infrared radiation (NIR), resulting in the generation of cytotoxic oxygen species (186). Since the EGFR-specific antibody mediates membrane proximity, only EGFR-positive cells are targeted by this approach. *In vitro*, Panitumumab-IR700 showed cytotoxic effects on EGFR-positive bladder cancer cells in a dose-dependent manner (183). Additionally, the effects are dependent on the expression level of EGFR and the applied NIR. As EGFR expression increases, lower NIR is needed to achieve similar IC50 values. In xenograft models, the bladder cancer cell line UMUC-5 was utilized to induce tumors that were subsequently treated with Panitumumab-IR700. NIR treated tumors showed regression, which was not observed in non-NIR-treated control groups. Furthermore, xenografts generated with cells exhibiting a lower EGFR-expression profile, showed no significant difference between NIR-treated and NIR-untreated animals, underlining the importance of antigen expression level on the tumor (183).

Since anti-tumor effects were limited by the surface expression of the antigen, Siddiqui and coworkers generated a combinatorial approach, consisting of Panitumumab-IR700 and Trastuzumab-IR700 (187). *In vitro* and *in vivo* experiments utilizing the EGFR/HER2 double-positive bladder cancer cell line SW-780 showed more potent effects upon combinatorial treatment in comparison to mono treatments (187). Even though there is no clinical study ongoing utilizing Panitumumab-IR700, the IR700 conjugated anti-EGFR PIT Cetuximab Salotarocan is currently under investigation in phase I/II studies for EGFR expressing advanced solid tumors in combination with anti-PD1 treatment, as well as phase III studies for head and neck cancer (NCT03769506). Under the name Acalux, Cetuximab Salotarocan was approved for the treatment of head and neck cancer in Japan in 2020.

## Radioimmunotherapy

The radioimmune approach combines the advantages of radiation therapy with the precision of immunotherapy by bringing a radioactive isotope in proximity to the tumor, mediated by the specific targeting of a mAb (188).

### ^213^Bi-Anti-EGFR-mAb

In 2009, Pfost and coworkers generated a conjugate of the anti-EGFR antibody Matuzumab and SCN-CHX-A”-diethylenetriaminepentaacetic acid (DTPA), resulting in a mAb-chelator conjugate (189, 190). Subsequently, the anti-EGFR-mAb was incubated with ^213^Bi, leading to chelation of the alpha-emitter by the conjugated mAb (**Figure 3**). The labeling yield of ^213^Bi to the mAb after 7 minutes was up to 97% and a specific activity of 0.35-1.4 MBq per µg antibody could be determined. Unbound ^213^Bi was separated by size exclusion chromatography. For *in vivo* experiments, mice were intravesical treated with luciferase transfected bladder carcinoma cells. Subsequently, mice were intravesical treated with ^213^Bi-Anti-EGFR-mAb, unlabeled mAb or the chemotherapeutic mitomycin c, respectively. Reaction to the treatment was analyzed utilizing bioluminescence. ^213^Bi-Anti-EGFR-mAb showed significantly higher anti-tumor activity compared to unlabeled mAb and did not show nephrotoxicity in contrast to mitomycin c (189).

In a subsequent study in 2015, the same group showed that application of even higher radiation doses did not lead to toxic side effects after instillation of ^213^Bi-Anti-EGFR-mAb in bladder cancer-bearing xenografts. Fractionated applications in advanced stage tumors were investigated, based on the idea, that an alpha-emitter is most potent when the diameter of the tumor does not exceed the emission range of the radioisotope. By applying multiple treatments, the tumor can be eradicated step-wise by eradicating the outer layer of the tumor in each treatment. It was demonstrated that various applications show a stronger potency *in vivo* (191). Still, radioimmunotherapeutic approaches for bladder cancer have not yet entered the clinic.

## Immunotoxins

Immunotoxins comprise the high toxicity and specificity of ADCs while bypassing the conjugation and heterogenicity problem by genetically fusing a targeting moiety (like an antibody) to a protein-based toxin (192).

### Oportuzumab Monatox

Oportuzumab Monatox is an immunotoxin, binding specifically to the epithelial cell adhesion molecule (EpCAM), which is a transmembrane glycoprotein, frequently overexpressed in different cancers (193), inter alia malignancies of the colon, intestine, breast, lung, and prostate (194).

Analysis of microarrays of high-grade and advanced stage bladder cancer tissues for EpCAM expression by IHC showed positive staining results in 54% of samples (195). EpCAM expression was linked to advanced stage, higher-grade tumors, and worse survival outcomes (195). This finding could be confirmed by another study performing enzyme-linked immunosorbent assay (ELISA) measurements utilizing the urine of bladder tumor patients (196). Nevertheless, EpCAM expression was no independent prognostic factor. It is hypothesized that EpCAM is involved in tumor progression, making it a potential immunotherapeutic target (195). The soluble variant of EpCAM, found in the urine, is generated by cleavage between A243 and G244 (196).

In 1997 Zimmermann and coworkers chemically conjugated a truncated *Pseudomonas aeruginosa* exotoxin A (ETA) variant (252–613) *via* thioether bonds to the EpCAM-specific, hybridoma derived mAb MOC31 (197). The lack of the ETA domain I, which mediates cell-binding, leads to an immunotoxin, which is only targeting EpCAM positive cells and showed potent cytotoxic effects *in vitro* and *in vivo*. Small and medium-sized tumors were either nearly completely eradicated or stopped tumor growth but failed to do so in larger tumors due to insufficient tumor penetration and limited dose-escalation due to unspecific toxicity (197). In the same year, Krebber and coworkers utilized the phage display technology to isolate the coding sequence of the hybridoma MOC31 (198). Even though its affinity was measured to be 3 nM, it failed to enrich at the tumor site in SW2 lung cancer xenografts (199). As it was hypothesized that this is due to its insufficient thermal stability, the antibody was humanized by grafting its CDR loops onto that Trastuzumab scFv 4D5 (199).

Furthermore, eight framework residues were transferred to generate a more stable antibody that exhibited parental specificity and affinity, resulting in enrichment in lung tumor xenografts (199). Paolo and coworkers further engineered the molecule by fusing it genetically C-terminal to a truncated *Pseudomonas aeruginosa* exotoxin A (ETA252-608) (200). In wild type ETA, amino acids 609-613, which was still used in the thiol conjugated immunotoxin (197), are encoding for the endoplasmic reticulum (ER) retention sequence REDLK. This sequence was altered to its mammalian counterpart, KDEL, which increases cytotoxicity (200, 201). The linker exhibited a furin cleavage site that allowed the release of the toxin in the endosome upon binding of the scFv to EpCAM and subsequent internalization (**Figure 3**). Two terminal His-Tags facilitate the purification of the immunotoxin after production in *E.coli*. As this construct showed potent and selective cytotoxicity *in vitro*, *in vivo* experiments were conducted, resulting in favorable anti-tumor effects (200).

After phase I/II studies in head and neck cancers, where the molecule resulted in a 88% response rate with 25% complete response, the renamed molecule (Oportuzumab Monatox) was subjected to local treatment in bladder cancer in a phase III study by intravesical administration twice weekly for six weeks, followed by six weeks with one administration per week (NCT02449239) (202). Enrolled patients were selected to suffer from NMIBC, especially CIS and/or exhibited high-grade Ta or T1 papillary disease and have not responded to previous BCG treatment. Additionally, a co-therapy phase I study is recruiting, combining Oportuzumab Monatox with the checkpoint inhibitor antibody Durvalumab (NCT03258593).

## mRNA-Based Protein Treatments

With the current SARS-CoV2 pandemic ongoing and the swift development of vaccine candidates, mRNA-based options are currently in the focus of the scientific community. Encoding for one or multiple proteins, the mRNA-based therapy enables the expression of the therapeutic entity within the patient, surpassing development issues of single proteins (203). Very recently, the first mRNA-based vaccines received emergency authorization to fight the SARS-CoV2 pandemic, potentially paving the way for mRNA-based therapeutics in the field of oncology.

### mRNA-2752

Formulated as a liquid nanoparticle encapsulated mRNA, mRNA-2752 encodes OX40L, IL-23, and IL-36γ, combining two different approaches: While IL-23 and IL-36γ are intended to act as adjuvants and to mediate a pro-inflammatory response within the tumor microenvironment (TME), the OX40 ligand activates T cells in order to generate an anti-tumor response (**Figure 3**). This, in turn, leads to the T cell antigen-dependent proliferation of CD4+ and CD8+ T cells and increases simultaneously their effector functions and the survival of memory T cells (204). In an ongoing phase I study, patients suffering from urothelial cancer and other malignancies are intratumorally injected with mRNA-2752 as monotherapy or in combination with Durvalumab (NCT03739931).

## CAR-T Cell Therapy

In recent years, transgenic T cells, exhibiting a chimeric antigen receptor (CAR), gained tremendous interest for cancer therapy. The concept dates back to the 1980s when an scFvs was fused to TCR in order to redirect the T cell specificity in a non-major histocompatibility complex (MHC)-restricted manner (205). CARs consist of an antibody-fragment, usually an scFv, that is genetically fused with CD3ζ (1^st^ generation) or additionally with the activation domain of CD28 or 4-1BB (2^nd^ generation). In 3^rd^ generation CARs, all three activation domains are implemented. 2^nd^ generation CAR T cells additionally equipped with transgene expression of cytokines or costimulatory ligand domains are referred to as 4^th^ generation (206). Upon antigen recognition, cytotoxic effects mediate target cell killing (207). Today, two CAR-T cell therapies are approved (Kymriah and Yescarta). While both target CD19, Kymriah is approved for refractory or relapse (R/R) B cell precursor acute lymphoblastic leukemia and Yescarta for R/R large B cell lymphoma (208, 209).

### 4SCAR-FRα/4SCAR-PSMA

Currently, two CAR-T cell approaches (**Figure 3**) targeting the folate receptor alpha (FRα) and the prostate-specific membrane antigen (PSMA) are in phase I/II studies for bladder cancer (NCT03185468). PSMA is significantly overexpressed in UCs and exhibits a stronger expression in NMIBC compared to MIBC, where its expression is correlated with recurrence and progression (210). FRα overexpression is often found in cancers of epithelial origin such as breast, ovary lung, or kidney cancer (211–213). In bladder cancer, FRα is significantly higher expressed in low-grade tumor tissue compared to high-grade tumors (214).

In these 4^th^ generation CAR-T cells, the targeting scFv is coupled to the costimulating intracellular domains of CD28, 4-1BB, CD27, and CD3ζ. Furthermore, as a “safety switch”, a suicide expression cassette was implemented consisting of the inducible caspase 9 (iCasp9) fused to FKBP12-F36V (215, 216). The latter can bind to the small molecule AP1903, resulting in homodimerization and activation of iCasp9. This way, activated CAR-T cells can be depleted by apoptosis in case of safety concerns (217). As CAR-T cell therapy showed poor performance in solid tumors (218), the result of this ongoing trial will illuminate the possibility of CAR-T therapy in the context of urethral cancers.

## Viral Gene Therapy

Gene therapy describes the delivery of genetic information to a host cell to introduce a therapeutic effect (219, 220). Besides genetic diseases, gene therapy has a strong focus on cancer therapy (221). As a promising tool for gene delivery, viral vectors were engineered to allow for a favorable safety and deliverability profile (222). As the bladder is a closed compartment, instillation of viral vectors can lead to a local treatment, which is not possible to achieve with systemic application of various drugs.

### CG0070

CG0070 is a cancer-selective adenovirus encoding for human GM-CSF (**Figure 3**). In an Ad5 backbone, the endogenous E1a promoter was replaced by the human E2F-1 promotor, which has a 5’ SV40 polyadenylation signal and promotes tumor-selective gene expression. Furthermore, the adenovirus exhibits the entire E3 region except for the gp19kD gene, which was replaced with the human GM-CSF gene under the control of the E3 promoter. The rest of the vector backbone, encoding for e.g., packaging signals, E2, E4 late protein regions, as well as inverted terminal repeat (ITR) sequences, were identical to the wild-type Ad5 (223).

As an oncolytic virus, CG0070 replicates selectively in malignant cells, resulting in the production of additional adenoviruses. Defects of the retinoblastoma (Rb) pathway are a shared trait of multiple cancers, including bladder cancer. The E2F-1 promoter, which controls the essential E1a gene, is selectively active in cells exhibiting an Rb pathway defect, resulting in its oncolytic characteristic. Furthermore, the production of the anti-tumoral GM-CSF is controlled by the E3 promoter, which in turn is dependent on the expression of the E1a gene product, resulting in E2F-1 dependent, and therefore tumor-selective, GM-CSF expression (223).

*In vitro* experiments confirmed the selectivity to Rb defective cells in terms of E1a and GM-CSF mRNA levels as well as Rb status dependent viral replication and cytotoxicity. Furthermore, *in vivo* experiments confirmed the anti-tumoral activity of CG0070 in bladder cancer xenografts, which showed synergistic effects in combination with docetaxel. However, these effects are due to the oncolytic effects of CG-0070 since human GM-CSF is not active in mice (223, 224). A first in-human study confirmed a favorable safety profile since no maximum tolerated dose was reached. The most common adverse event was grade 1-2 bladder toxicity.

Additionally, high doses of GM-CFS were observed in the urine of patients. CG0070 treatment resulted in a complete response rate of 48.6% for a median duration of 10.4 months (NCT00109655) (225). Intravesical administration of CG-0070 was established in a subsequent phase II study after prior bladder rise with saline solution and 0.1% dodecylmaltoside (DDM) to enhance transduction efficiently. The interim report described a complete response in 47% of all patients after six months and 50% in CIS patients while exhibiting an acceptable toxicity profile (NCT02365818) (226). Enrolled patients failed intravesical BCG treatment 12 to 24 months prior to CG0070 treatment, thus patients are not BCG unresponsive as per FDA definition. Currently, a phase II study of CG0070 in combination with checkpoint inhibitor Pembrolizumab is ongoing (NCT04387461), as well as a phase III study of CG0070 as a monotherapy (NCT04452591). Both studies focus on the treatment of NMIBC.

### rAd-IFN/Syn-3 (Adstiladrin/Nadofaragene Firadenovec)

In 2001 Iqbal Ahmed and coworkers generated a recombinant replication-deficient adenovirus carrying the genetic information for human interferon-α2b under the control of a CMV promoter (**Figure 3**) (227). This constructed rAdIFNα2b viral vector revealed significant anti-tumor activity and suppressed primary and metastatic tumor growth *in vivo* (227). These findings reflect previous findings, which demonstrated that adenoviral-based interferon-assisted immunotherapy yields promising results (228, 229).

A major obstacle for viral gene therapy in the context of bladder cancer was the glycosaminoglycan layer (GAG), which shields the surface of urothelial cells from viral infection and hindered effective transduction (230, 231). To overcome this problem, Connor and coworkers identified polyamides that enable efficient viral transduction *in vivo* (232). Further bioanalytical characterization revealed that one impurity of the initial heterogenic detergent was the bioactive compound (231, 232). Subsequent chemical modification led to the generation of Syn-3, which mediated an enhanced *in vivo* transduction efficacy in the bladder while exhibiting a favorable safety profile (231, 233).

In 2004 Benedict and coworkers combined the adenoviral IFN approach with Syn3 and performed intravesical instillation in mice, resulting in high urinary IFN levels for at least seven days, while intravesical instillation of rIFN protein alone was detectable for only 12h. Regression of GFP-labeled tumor cells was detected after 1h treatment to rAd-IFN/Syn-3, while no cytotoxic effect on normal tissue was observed. Even cell lines previously unresponsive to rIFNα treatment showed cytotoxic effects *in vitro* and *in vivo* (234). Intravesical instillation of IFN alone or the rAd-IFN without Syn3 showed no impact on tumor burden.

A phase I study evaluating the safety and efficacy showed no dose-limiting toxicities (NCT01162785). Except for patients receiving the lowest dose, effective gene transfer was found in all patients with detectable IFNα levels. 43% of the enrolled patients experienced a complete response at three months, where two remained disease-free at month 29 and 39, respectively (235). The subsequent phase II study enrolling high-grade BCG refractory or relapsed NMIBC patients showed that 35% of patients achieved 12 month without high-grade recurrence (NCT01687244) (236). Very recently, the primary efficacy analysis of a phase III study in BCG-unresponsive NMIBC patients was published (NCT02773849), where 53.4% of enrolled CIS patients had a complete response at 3 months and 45.5% of those maintained this outcome at 12 months (237). The mechanism of action of Nadofaragene Firadenovec is depicted in **Figure 2D**.

## Discussion

A variety of engineered antibodies and proteins are in preclinical development and clinical trials for the treatment of BC. Among them, checkpoint inhibitors were the first approved biologics for bladder cancer, but the list is likely to be extended. In particular, ADCs are promising entities to have a major impact on the treatment of bladder cancer in the future (128). By combining the specificity of an antibody with the strong cytotoxic effect of its payload, ADCs circumvent systemic exposure of the toxin and specifically target the tumor. Engineering of the linkers to be cleaved by endoplasmic enzymes or pH-dependent release of the payload further contributes to the efficacy and safety of this drug class. Enfortumab Vedotin was recently approved for BC, but the list of approved ADCs will most probably expand in the near future. While Trastuzumab Deruxtecan and Sirtratumab Vedotin are currently in phase I studies, phase II studies are ongoing for Disitamab Vedotin, ado-Trastuzumab Emtansine, and Sacituzumab Govitecan investigating efficacy in bladder cancer patients (**Table 1**).

Bispecific antibodies and fusion proteins are also next generation biologics and are under investigation for bladder cancer. Even though exhibiting interesting molecular designs, most bispecifics are in early stages of clinical development (**Table 1**). While preclinical assessment of T cell engagers like Orlotamab, Treg depleting bsAb like ATOR-1015 or super agonistic cytokine traps like ALT-803 exhibit promising profiles, only ongoing and future trials will show whether these classes of molecules will have an impact in uro-oncology.

However, ADCs, bispecifics, and most fusion proteins are applied systemically and despite the specific tumor targeting mediated by the binding moiety, systemic side effects and resulting dose-limitations may accrue. Furthermore, molecule heterogeneity remains a challenge for ADC development. The immunotoxin Oportuzumab Monatox circumvent both problems by genetic fusion of the bioactive drug ETA to the scFv and local administration to the bladder *via* instillation. As clinical trials utilizing this molecule resulted in encouraging results (**Table 1**), a rolling biologics license application (BLA) submission is currently under review by the FDA, which might lead to the FDA approval of Oportuzumab Monatox.

The instillation of Oportuzumab Monatox results in a local treatment of the tumor but also in a shorter half-life. This limitation is circumvented by gene therapeutic approaches, leading to a prolonged local treatment. CG0070 and rAd-IFN/Syn-3 are adenovirus-based vectors targeting the bladder tissue and lead to the production of anti-tumor cytokines upon transduction. While CG0070 does this in a non-tumor-selective manner, resulting in an oncolytic mechanism of action, rAd-IFN/Syn-3 transduces cells in a non-tumor selectivemanner, leading to sustained high IFNα levels and a subsequent convenient quarterly treatment schedule with an excellent safety profile, where only 1.9% of the patients discontinued treatment. A phase III study for CG0070 is still ongoing, while the results of the phase III study of rAd-IFN/Syn-3 recently showed very promising outcomes, paving the way for a potential future approval (237). rAd-IFN/Syn-3 was granted priority review, fast track and breakthrough therapy designations by the FDA with the BLA currently under investigation.

Of note, the clinical outcomes of CG0070 and rAd-IFN/Syn-3 are not directly comparable as the patient populations are not identical. The phase II study of CG0070 enrolled patients described as BCG-unresponsive but allowed for patients with disease recurrence up to 24 months to be included and did not solely request patients to have received adequate prior BCG treatment according to the BCG Unresponsive criteria (NCT02365818). By now, BCG unresponsive patients must show recurrence within 12 months for CIS, or 6 months for Ta/T1, or 3 months for T1 to be defined as BCG-unresponsive and depicts a population very unlikely to respond to further BCG treatment (20, 21). The patient in the GC0070 phase II study most likely had a lower risk of recurrence/progression compared to the population of rAd-IFN/Syn-3 trial (NCT01687244) and might have shown a positive response to other instillation treatments, including rechallenge with BCG compared to a true BCG-unresponsive population. Additionally, the dosing scheme of rAd-IFN/Syn-3 is less time consuming and more convenient compared to CG0070. Therefore, it is tempting to speculate that rAd-IFN/Syn-3 has a better stand in the direct comparison and might be the favorable treatment of choice. In respect to all these information, Kulkarni very recently elaborated the potential of rAd-IFN/Syn-3 to become the “New Gold Standard” for BCG-unresponsive bladder cancer patients (238).

Due to the ongoing SARS-CoV2 pandemic, mRNA-based medicine is in the spotlight. Due to its intratumoral application, mRNA-2752 is locally active in the tumor of the patient, where the expression of immunoregulatory proteins might lead to a strong anti-tumor effect. Furthermore, mRNA-based therapies are more straightforward to develop compared to virus-based therapeutics. Even though mRNA-based vaccines have recently gained emergency use authorization, the clinical studies of mRNA-based therapeutics for cancer treatment are still in early testing.

Next-generation molecules like ADCs, immunotoxins, and gene therapeutic approaches will most probably change the way bladder cancer is treated. Conventional antibodies, on the other hand, except checkpoint inhibitors, will probably not have a major impact on UC treatment. Vofatamab, an anti-FGFR3 antibody, was in three phase I and phase I/II studies for bladder cancer, all of which were terminated, even though FGFR3 seems to be an interesting target, especially for NMIBC.

For the development of future therapeutics, a special focus will be set on the route of administration. As the bladder is relatively uncomplicatedy to reach from the outside and local treatments will result in less systemic side effects, future molecules will focus on application by instillation. Oportuzumab Monatox demonstrates that this is a feasible route to administer biologics. In line with current events, mRNA- and viral-based therapeutics will play a tremendous role in the coming years and will most probably have a major impact on pharmaceutical research, leading to new molecules with novel mechanisms of action.

Taken together, there is a broad pipeline of molecules exhibiting different design strategies and various mechanisms of action. From stimulating the immune system within the TME, recruiting of effector cells to directly or indirectly kill cancerous cells, several therapeutic approaches are under clinical testing for bladder cancer, giving hope to patients in need. Future approval of ADCs, immunotoxins, and gene therapeutic approaches will have a strong impact on bladder cancer treatment. These additional options are expected to result in beneficial clinical outcomes and less severe side effects compared to present-day treatments.

## Author Contributions

JB, BH, and HK conceived this review. JB wrote the first draft of the manuscript. JB, JJ, BH, and HK wrote sections of the manuscript. JG and AB gave scientific advice. All authors contributed to the article and approved the submitted version.

## Funding

This work was supported by the Ferring Darmstadt Labs at Technical University of Darmstadt and by the department of GPRD at Ferring Holding S.A., Saint-Prex. The funder bodies were not involved in the study design, collection, analysis, interpretation of data, the writing of this article or the decision to submit it for publication.

## Conflict of Interest

JG, JJ, AB, and BH were employed by the company Ferring Pharmaceuticals. JB is employed by TU Darmstadt in frame of a collaboration project with Ferring Pharmaceuticals.

The remaining author declares that the research was conducted in the absence of any commercial or financial relationships that could be construed as a potential conflict of interest.
